# Toll-like receptor 3 upregulation by type I interferon in healthy and scleroderma dermal fibroblasts

**DOI:** 10.1186/ar3221

**Published:** 2011-01-11

**Authors:** Sandeep K Agarwal, Minghua Wu, Christopher K Livingston, Donald H Parks, Maureen D Mayes, Frank C Arnett, Filemon K Tan

**Affiliations:** 1 Division of Rheumatology and Clinical Immunogenetics, Department of Internal Medicine, The University of Texas Health Science Center at Houston, 6431 Fannin Avenue, Houston, TX 77030, USA; 2 Division of Plastic and Reconstructive Surgery, Department of Surgery, The University of Texas Health Science Center at Houston, 6431 Fannin Avenue, Houston, TX 77030, USA

## Abstract

**Introduction:**

Increased levels of genes in the type I interferon (IFN) pathway have been observed in patients with systemic sclerosis (SSc), or scleroderma. How type I IFN regulates the dermal fibroblast and its participation in the development of dermal fibrosis is not known. We hypothesized that one mechanism by which type I IFN may contribute to dermal fibrosis is through upregulation of specific Toll-like receptors (TLRs) on dermal fibroblasts. Therefore, we investigated the regulation of TLR expression on dermal fibroblasts by IFN.

**Methods:**

The expression of TLRs was assessed in cultured dermal fibroblasts from control and SSc patients stimulated with IFNα2. The ability of IFNα2 to regulate TLR-induced interleukin (IL)-6 and CC chemokine ligand 2 production was also assessed. Immunohistochemical analyses were performed to determine whether TLR3 was expressed in skin biopsies in the bleomycin-induced skin fibrosis model and in patients with SSc.

**Results:**

IFNα2 increased TLR3 expression on human dermal fibroblasts, which resulted in enhanced TLR3-induced IL-6 production. SSc fibroblasts have an augmented TLR3 response to IFNα2 relative to control fibroblasts. Pretreatment of fibroblasts with transforming growth factor (TGF)-β increased TLR3 induction by IFNα2, but coincubation of TGF-β did not alter TLR3 induction by IFN. Furthermore, IFNα2 inhibits but does not completely block the induction of connective tissue growth factor and collagen expression by TGF-βin fibroblasts. TLR3 expression was observed in dermal fibroblasts and inflammatory cells from skin biopsies from patients with SSc as well as in the bleomycin-induced skin fibrosis model.

**Conclusions:**

Type I IFNs can increase the inflammatory potential of dermal fibroblasts through the upregulation of TLR3.

## Introduction

Systemic sclerosis (SSc), or scleroderma, is a multisystem autoimmune disease clinically characterized by progressive fibrosis of the skin and internal organs. Pathologically, SSc exhibits three cardinal features: inflammation and autoimmunity, vasculopathy and excessive extracellular matrix (ECM) deposition [1]. The ECM consists of collagens, proteoglycans, fibrillins and other matrix molecules [2]. Located within this matrix are fibroblasts and myofibroblasts, key effectors of the fibrotic process. Resident and infiltrating cells in the dermis secrete soluble mediators, such as transforming growth factor β (TGF-β), that activate fibroblasts and induce differentiation into myofibroblasts [3,4]. The myofibroblasts subsequently produce large amounts of ECM, leading to fibrosis. In addition to their role in ECM deposition, dermal fibroblasts and myofibroblasts are capable of secreting inflammatory cytokines and chemokines, such as interleukin (IL)-6 and CC chemokine ligand 2 (CCL-2), important inflammatory mediators in SSc pathogenesis [5-8]. Thus, fibroblasts also may contribute to the development of dermal fibrosis through the production of these inflammatory mediators.

Current paradigms point toward systemic immune dysregulation as a central process that ultimately may lead to fibroblast activation. Biopsies of early SSc skin demonstrate perivascular infiltrates of mononuclear inflammatory cells, which produce cytokines and chemokines that recruit inflammatory cells and promote ECM deposition [9]. More recent studies in patients with SSc have identified dysregulation of type I interferon (IFN) pathways similar to those seen in patients with systemic lupus erythematosus (SLE) [10-12]. Gene expression profiling of peripheral blood has demonstrated the presence of a type I IFN signature in patients with SSc [12]. These findings have been confirmed in both circulating CD14^+ ^monocytes and CD4^+ ^T-cells, as well as in skin biopsies from patients with SSc compared with healthy controls [13-15]. Together these data demonstrate the presence of a type I IFN signature in circulating blood cells and a major target organ (skin) in patients with SSc.

Type I IFNs are potent regulators of the immune system, where they modulate the differentiation, survival, proliferation and cytokine production of T-cells, B-cells and dendritic cells. Among the critical immunoregulatory functions of IFN is its ability to stimulate the expression of Toll-like receptors (TLRs) on dendritic cells. TLRs are a family of germ line-encoded proteins that serve as pattern recognition receptors capable of recognizing highly conserved motifs present in infectious microorganisms called pathogen-associated molecular patterns (PAMPs) [16]. While their roles are best characterized on antigen-presenting cells, various TLRs also are expressed on fibroblast populations [17,18]. Interestingly, IFN increases TLR3 and TLR7 expression on fibroblast-like synoviocytes (FLS) and enhances TLR-induced inflammatory cytokine production by FLS [18].

Given the reported influence of IFN on FLS and the importance of dermal fibroblasts in the pathogenesis of SSc, it is important to understand how IFN may modulate the dermal fibroblast. We hypothesized that one mechanism by which type I IFN may contribute to the pathogenesis of SSc is through upregulation of the expression of specific TLRs on dermal fibroblasts.

## Materials and methods

### Reagents

Recombinant human TGF-β and IFNα2 were purchased from eBioscience Inc. (San Diego, CA, USA). TLR agonists Pam3CysK4; polyinosinic:polycytidylic acid, or poly(I:C); lipopolysaccharide (LPS) and Gardiquimod ([1-(4-amino-2-ethylaminomethylimidazo[4,5-c]quinolin-1-*yl*)-2-methylpropan-2-ol]) were purchased from InvivoGen (San Diego, CA, USA).

### Fibroblast cultures

Skin biopsy specimens of clinically uninvolved skin were obtained from patients with SSc and from control patients without a history of autoimmune disease. All patients with SSc fulfilled the American College of Rheumatology criteria for SSc [19]. All patients provided written consent, and the study was approved by the Committee for the Protection of Human Subjects at the University of Texas Health Science Center at Houston.

Dermal fibroblast cultures were isolated as previously described [20]. Cultured fibroblast strains were established by mincing tissues and placing them into 60-mm culture dishes secured by glass coverslips. The primary cultures were maintained in Dulbecco's modified Eagle's medium (DMEM), 10% fetal bovine serum (FBS), 2 mM L-glutamine, 100 U/mL penicillin, and 50 μM 2-mercaptoethanol at 37°C with 5% CO_2_. Passages 4-8 dermal fibroblasts were used for experiments.

### RNA isolation and quantitative real-time polymerase chain reaction

Fibroblasts (3 × 10^4^) were cultured in 100 μL DMEM with 10% FBS in 96-well plates overnight. Cultures were subsequently rested overnight in DMEM with bovine serum albumin (BSA), then stimulated with cytokines in DMEM with BSA for 24 hours. Total RNA was isolated and cDNA was synthesized using the TaqMan Gene Expression Cells-to-CT™ Kit (Applied Biosystems Inc., Foster City, CA, USA). Quantitative real-time PCR (qRT-PCR) was performed using validated TaqMan Gene Expression assays for human TLR2 (Hs00152973_m1), TLR3 (Hs01551078_m1), TLR4 (Hs01060206_m1), TLR7 (Hs00152971_m1), TLR9 (Hs00152973_m1), connective tissue growth factor (CTGF) (Hs00170014_m1) and cyclophilin (Hs99999904_m1) (Applied Biosystems Inc.) on an Applied Biosystems 7900HT Fast Real-Time PCR System. Cyclophilin was used as an endogenous control to normalize transcription levels of total RNA in each sample. The data were analyzed using SDS 2.3 software (Applied Biosystems Inc., Foster City, CA, USA) and the comparative CT method (2-^ΔΔ^^*C*^_T _method). The fold change was calculated as 2-^ΔΔ^^*C*^_T_.

### Cytokine production

Fibroblasts (3 × 10^5^) were cultured in 1 ml DMEM with 10% FBS in 24-well plates overnight. Cultures were subsequently rested overnight in DMEM with BSA, then stimulated with TLR agonists (10 μg/mL) in DMEM with BSA for 48 hours. Supernatants were harvested and frozen at -80°C. IL-6 and CCL-2 levels were determined by performing enzyme-linked immunosorbent assay (eBioscience, Inc.).

### Bleomycin dermal fibrosis mouse model

Six- to eight-week-old female C57BL/6 mice (Jackson Laboratory, Bar Harbor, ME, USA) were used in these studies. The protocols were approved by the University of Texas Health Science Center at Houston Animal Care and Use Committee. Filter-sterilized bleomycin 0.02 U per mouse was dissolved in phosphate-buffered saline (PBS) (Teva Parenteral Medicines, Irvine, CA, USA), or PBS was administered by daily subcutaneous injections for 28 days into the shaved backs of mice using a 27-gauge needle. At the end of the experiment, mice were humanely killed and lesional skin was processed for analysis.

### Immunohistochemistry

Skin biopsies were obtained from four patients with SSc and from four healthy controls without a known history of autoimmune disease from the National Disease Research Interchange (Philadelphia, PA, USA). Five-micrometer sections were deparaffinized, rehydrated and immersed in Tris-buffered saline and 0.1% Tween 20, then treated with target retrieval solution (Dako, Carpinteria, CA, USA) at 95°C for 10 minutes. Rabbit polyclonal primary antibodies against TLR3 or an isotype-matched control antibody (Abcam Inc., Cambridge, MA, USA) were used. Bound antibodies were detected using secondary antibodies from the Dako Cytomation Envision System-HRP (3,3-diaminobenzidine tetrahydrochloride). Sections were counterstained with hematoxylin.

### Statistical analysis

Data were imported into GraphPad Prism software for graphing and analysis (GraphPad Software, Inc., La Jolla, CA, USA). Data are given as means, and error bars represent the standard error of the mean. Nonparametric paired (Mann-Whitney *U *test) and unpaired (Wilcoxon signed-rank test) *t*-tests were used when appropriate.

## Results

### TLR3 upregulation by IFN-α2 in cultured dermal fibroblasts

**Figure 1:**
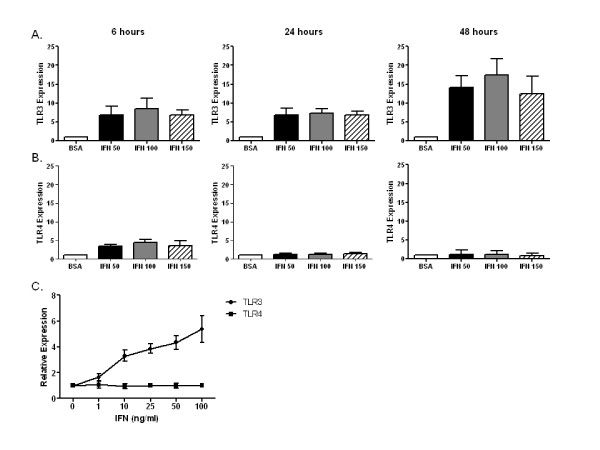
**Toll-like receptor 3 (TLR3) upregulation by interferon α (IFNα)**. Dermal fibroblasts from healthy control skin were cultured *in vitro *with IFNα (50-150 ng/mL) or 0.1% bovine serum albumin (BSA) for 6, 24 and 48 hours. Total RNA was harvested, and **(A) **TLR3 and **(B) **TLR4 mRNA levels were determined by performing quantitative real-time polymerase chain reaction (qRT-PCR) assays. IFN induced TLR3 upregulation at 6, 24 and 48 hours. TLR4 upregulation was noted only at 6 hours. **(C) **Dose-response curve for TLR3 upregulation by IFNα (0-100 ng/mL) for 24 hours in healthy control dermal fibroblasts. n = 3 control cell lines.

Dermal fibroblasts from controls were stimulated with media or human recombinant IFNα2 for 24 hours. Total RNA was isolated and qRT-PCR was performed to determine the relative expression of TLR2, TLR3, TLR4, TLR7, TLR8 and TLR9. As shown in Figure 1A, TLR3 expression was upregulated by IFNα2 (50-150 ng/mL) at 6 hours and remained elevated at 24 and 48 hours. In contrast, TLR4 expression was slightly upregulated by IFNα2 at 6 hours, but at 24 and 48 hours no change in TLR4 expression was observed compared with dermal fibroblasts cultured in media alone. Expression of TLR2, TLR7, TLR8 and TLR9 was below the limits of detection (data not shown). Additional experiments demonstrated that TLR3 but not TLR4 expression was upregulated in a dose-dependent fashion (Figure 1B), with a concentration as little as 1 ng/mL IFNα2 stimulating the expression of TLR3. These data clearly demonstrate the upregulation of TLR3 expression by IFNα2 in control dermal fibroblasts.

**Figure 2:**
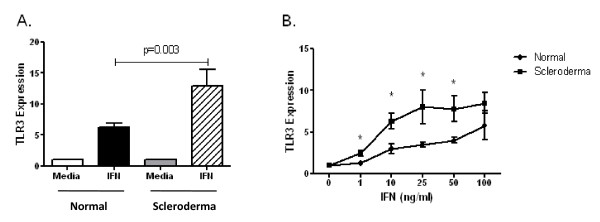
**Comparison of TLR3 upregulation by IFNα in healthy control and systemic sclerosis (SSc), or scleroderma, dermal fibroblasts**. **(A) **Dermal fibroblasts were stimulated for 24 hours with 50 ng/mL IFNα, and TLR3 was determined by performing qRT-PCR assays. The magnitude of induction of TLR3 expression by IFNα was significantly greater in dermal fibroblasts from patients with SSc (n = 11) than in those from healthy controls (n = 25; *P *= 0.003). **(B) **SSc dermal fibroblasts have a greater magnitude of upregulation of TLR3 with IFN at concentrations ranging from 1 to 100 ng/mL (n = 4 in each group; *P < 0.05 (Wilcoxon signed-rank test)).

The upregulation of TLR3 expression by IFNα2 was compared between SSc and control dermal fibroblasts. The magnitude of induction of TLR3 expression by IFNα2 was significantly greater in dermal fibroblasts from patients with SSc than in controls (Figure 2A). This increase in TLR3 expression was observed when dermal fibroblasts were stimulated with IFNα2 at concentrations from 1 to 100 ng/mL, although at 100 ng/mL the difference was not statistically significant (Figure 2B). These data demonstrate that SSc cultured fibroblasts have a greater magnitude of upregulation of TLR3 by IFNα2 than that of control fibroblasts.

### IFNα2 increases TLR3-induced IL-6 production in cultured dermal fibroblasts

**Figure 3:**
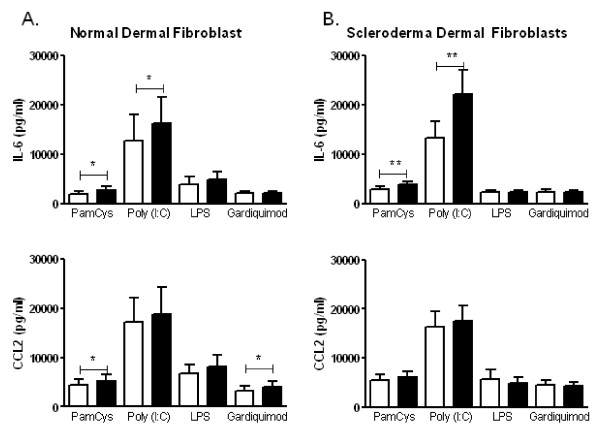
**IFN increases TLR3-induced interleukin (IL)-6 production in cultured dermal fibroblasts**. **(A) **Healthy control fibroblasts (n = 10) and **(B) **SSc dermal fibroblasts (n = 10) were preincubated with media alone or with 50 ng/mL IFNα for 24 hours, washed and then stimulated with Pam_3_CysK_4 _(TLR2 agonist); polyinosinic:polycytidylic acid, or poly(I:C) (TLR3 agonist); lipopolysaccharide (TLR4 agonist) and Gardiquimod (TLR7/8 agonist; [1-(4-amino-2-ethylaminomethylimidazo[4,5-c]quinolin-1-*yl*)-2-methylpropan-2-ol]) for 48 hours (10 μg/mL). Culture supernatants were assessed for IL-6 and CC chemokine ligand 2 (CCL2). Preincubation with IFNα increased poly(I:C)-stimulated IL-6 but not CCL2 production from healthy control and SSc dermal fibroblasts. *P < 0.05, **P < 0.01 (Wilcoxon signed-rank test).

To determine whether the upregulation of TLR3 mRNA resulted in changes in functional TLR levels, dermal fibroblasts were preincubated with media alone or with 50 ng/mL IFNα2 for 24 hours. Cultures were subsequently stimulated with a panel of TLR agonists, and cytokine and chemokine production were assessed. Pam_3_CysK_4 _(a TLR2 agonist), poly(I:C) (a TLR3 agonist), LPS (a TLR4 agonist) and Gardiquimod (a TLR7/8 agonist) were all used at 10 μg/mL (Figure 3A).

Culture supernatants from control dermal fibroblasts stimulated with the TLR3 agonist poly(I:C) produced high levels of IL-6 and CCL-2. Preincubation of dermal fibroblasts with IFNα2 resulted in increased IL-6 production (*P *= 0.01) but not CCL-2 production compared with dermal fibroblasts preincubated with BSA. Consistent with the qRT-PCR data shown in Figure 1, preincubation with IFNα2 did not significantly increase TLR4-induced production of IL-6 or CCL-2. Last, while IFNα2 preincubation slightly increased the levels of IL-6 and CCL-2 in cultures stimulated with TLR2 or TLR7/8 agonists, these levels were not higher than those of unstimulated dermal fibroblasts (data not shown). These data suggest that IFNα2 preincubation results in enhanced IL-6 production to the TLR3 agonist poly(I:C).

SSc dermal fibroblasts also demonstrated enhanced IL-6 production to the TLR3 agonist poly(I:C), but not to other TLR agonists. In Figure 3B, the level of IL-6 in culture supernatants from cells preincubated with IFNα2 followed by TLR3 stimulation with poly(I:C) was significantly higher than that in SSc dermal fibroblasts preincubated in media alone followed by poly(I:C) stimulation (*P *= 0.002). In contrast, IFNα2 preincubation did not significantly increase poly(I:C)-induced production of CCL-2. The IL-6 production in TLR2-stimulated cultures was not higher than that in media alone (data not shown). These data demonstrate that IFNα2 specifically upregulates TLR3 expression in dermal fibroblasts, which results in increased IL-6 production upon TLR3 stimulation of dermal fibroblasts.

### Myofibroblasts have increased upregulation of TLR3

SSc skin biopsies have increased numbers of myofibroblasts [3]. *In vitro *TGF-β induces the differentiation from fibroblasts to myofibroblasts [21]. Since SSc fibroblasts have an increased induction of TLR3 by IFNα2 compared with control fibroblasts, we sought to determine whether IFNα2 induction of TLR3 expression was increased in myofibroblasts.

**Figure 4:**
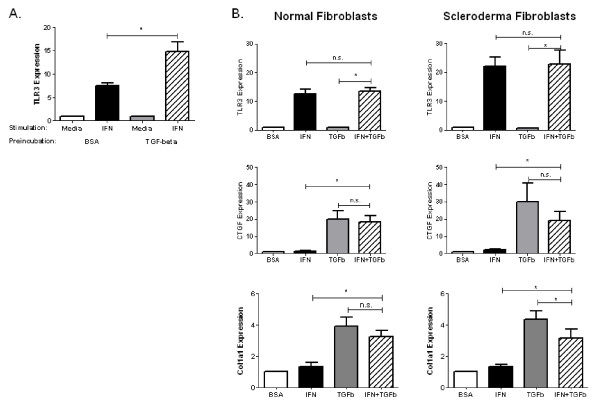
**Cross-regulation of IFNα and TGFβ in dermal fibroblasts**. **(A) **Healthy control dermal fibroblasts were cultured in 10 ng/mL TGFβ for 72 hours to induce myofibroblast differentiation *in vitro*. After 72 hours, cultures were washed and subsequently stimulated with 50 ng/mL IFN for 24 hours. Total RNA was analyzed for TLR3 by qRT-PCR assay. Preincubation with TGFβ resulted in a greater induction of TLR3 by IFN compared with fibroblasts preincubated in 0.1% BSA (n = 7; *P *= 0.02). **(B) **Dermal fibroblasts were incubated with 50 ng/mL IFN, 10 ng/mL TGF-β or both cytokines for 24 hours. Total RNA was analyzed for TLR3, connective tissue growth factor (CTGF), and collagen type I, α_1 _(COL1A1) expression by qRT-PCR assay. Coincubation of fibroblasts with IFN and TGF-β did not alter the expression of TLR3 compared with IFN alone. IFN did not alter TGF-β-induced CTGF expression but did slightly reduce COL1A1 expression in SSc dermal fibroblasts. n = 7, *P < 0.05, n.s. = not significant (Wilcoxon signed-rank test).

Control dermal fibroblasts were cultured in TGF-β for 72 hours to induce myofibroblast differentiation *in vitro*, followed by stimulation with IFNα2 for 24 hours. As expected, TGF-β increased the number of cultured fibroblasts expressing α-smooth muscle actin as detected using immunofluoresence (data not shown). Interestingly, dermal fibroblasts preincubated with TGF-β had greater induction of TLR3 by IFNα2 compared with fibroblasts preincubated in media alone (14.83 ± 2.06 vs. 7.46 ± 1.62; *P *= 0.02) (Figure 4A). In contrast, dermal fibroblasts preincubated with TGF-β had a decrease in TLR4 induction by IFNα2 compared with fibroblasts preincubated in media alone (1.1 ± 0.1 vs. 1.6 ± 0.1; *P *= 0.001). Therefore, myofibroblasts display increased upregulation of TLR3 in response to IFNα2.

### Coincubation of IFNα2 and TGF-β

Multiple lines of evidence point to the dysregulation of TGF-β and IFNα2 in SSc [12,22]. How these two cytokines interact at the level of the dermal fibroblasts has not been fully elucidated. TGF-β has profibrotic properties, while previous studies have suggested that IFN may have antifibrotic properties. It is reasonable to hypothesize that dermal fibroblasts might be exposed simultaneously to both IFNα2 and TGF-β *in vivo*. Therefore, we next sought to ascertain the effects of the IFNα2-induced TLR3 upregulation during simultaneous exposure to TGF-β.

Fibroblasts were incubated with IFNα2, TGF-β or both cytokines for 24 hours. Total RNA was harvested for qRT-PCR analysis (Figure 4B). TLR3 expression was increased by IFNα2 in both control and SSc fibroblasts. Coincubation of fibroblasts with IFNα2 and TGF-β did not change the expression of TLR3 compared with IFNα2 alone. CTGF and type I collagen expression also were assessed to determine whether concentrations of IFNα2 that induced TLR3 have antifibrotic properties. CTGF expression was increased by TGF-β in both control and SSc fibroblasts (20.04 ± 4.6 and 30.13 ± 10.62, respectively). IFNα2 resulted in a slight nonsignificant decrease in TGF-β-stimulated CTGF expression in both control and SSc fibroblasts (18.27 ± 3.9 and 19.17 ± 2.58, respectively). Furthermore, collagen, type I, α_1 _(COL1A1) expression was increased by TGF-β in both healthy control and SSc fibroblasts (3.90 ± 0.60 and 4.34 ± 0.58, respectively). IFNα2 resulted in a slight decrease in COL1A1 expression in both control and SSc fibroblasts; however, this difference was significant only in the SSc fibroblasts (3.25 ± 0.41 and 3.13 ± 0.58, respectively). The expression of CTGF and COL1A1 was significantly higher in dermal fibroblasts stimulated with both IFNα2 and TGF-β compared with media or IFNα2 alone, suggesting that IFNα2 only blunted the TGF-β induction of CTGF and COL1A1. These data suggest that IFNα2 may decrease expression of matrix-related genes important in the development of dermal fibrosis; however, at concentrations that induce TLR3 expression, the magnitude of inhibition is relatively small compared with the overall induction by TGF-β alone.

#### TLR3 expression in fibrotic and scleroderma skin

**Figure 5:**
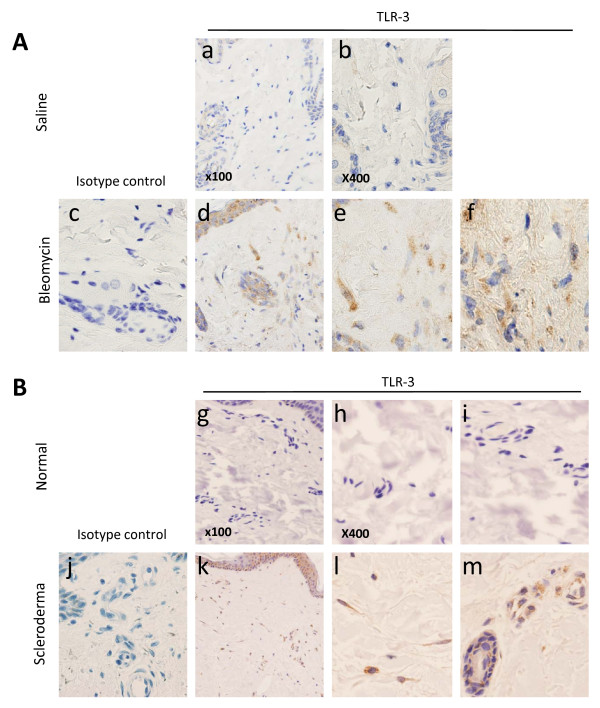
**Immunohistochemical analyses of TLR3 expression in dermal fibrosis**. Immunohistochemical analyses were performed using rabbit polyclonal antibodies against TLR3 (histograms a, b, d-f, g-i, k-m) or isotype control (histograms c and j). **(A) **Skin biopsies from mice injected with bleomycin, but not saline, demonstrated expression of TLR3 in the dermis (panel d), which localized to fibroblast-like cells (histogram e) and inflammatory cells (histogram f). n = 3 saline, n = 3 bleomycin. **(B) **Skin biopsies from control skin (n = 4) and SSc skin (n = 4) demonstrated TLR3 expression in the dermis of SSc skin (histogram k), which localized to fibroblast-like cells and inflammatory cells (histogram l) as well as to endothelial cells (histogram m) in SSc but no control skin.

The data above were obtained using cultured dermal fibroblasts. To determine whether TLR expression is also found in the fibroblasts *in vivo*, immunohistochemical studies were performed to localize the expression of TLR3 in skin from the bleomycin-induced skin fibrosis model (Figure 5A), as well as from the skin biopsies of healthy controls and patients with SSc (Figure 5B).

Skin biopsies were performed on mice injected daily for 28 days with subcutaneous saline or bleomycin. Staining with an antibody specific for TLR3 did not reveal any detectable level of TLR3 expression in saline-injected skin (Figure 5A, histograms a and b). In contrast, skin biopsies from mice injected with bleomycin demonstrated expression of TLR3 that was present in cells of the dermis (Figure 5A, histogram d), which localized to fibroblast-like cells (Figure 5A, histogram e) as well as some inflammatory cells (Figure 5A, histogram f). These data demonstrate that TLR3 expression is increased in the dermis of mice injected with bleomycin.

To determine whether TLR3 is expressed in human skin, immunohistochemistry was performed for TLR3 in healthy control skin biopsies and SSc skin biopsies. TLR3 expression was not detectable in the dermis of healthy control skin (Figure 5B, histograms g-i). In contrast, TLR3 expression was observed with higher-power magnification in the dermis of SSc skin (Figure 5B, histogram k) which was localized to fibroblast-like cells as well as inflammatory cells (Figure 5B, histogram l). Last, in SSc skin, the endothelial cells also demonstrated expression of TLR3 (Figure 5B, histogram m), which was not observed in healthy control skin biopsies. Therefore, similar to the *in vitro *data, TLR3 is expressed on fibroblasts in SSc biopsies.

## Discussion

In the current article, we have demonstrated that IFNα2, a type I interferon, increases the expression of TLR3 on human dermal fibroblasts, which results in enhanced TLR3-induced IL-6 production. Dermal fibroblasts from patients with SSc have an augmented response to IFN with regard to TLR3 expression. Consistent with the *in vitro *data, we also have demonstrated that skin biopsies from patients with SSc as well as the bleomycin-induced skin fibrosis model both have TLR3 expression that localizes to fibroblast-like cells. Importantly, pretreatment with TGF-β increased TLR3 induction by IFNα2, but coincubation of TGF-β does not alter TLR3 induction by IFNα2. Last, IFNα2 inhibits but does not completely block the induction of CTGF and collagen expression by TGF-β in dermal fibroblasts.

TLR3 is a member of the TLR family that recognizes double-stranded RNA, which is a molecular pattern produced by many viruses at some point in their infectious cycle [17]. TLR3 is expressed on endosomes of dendritic cells, but has been reported on the cell surface as well as in endosomes of fibroblasts [17]. Activation of TLR3 results in the production of type I IFN, which may in turn further upregulate the expression of TLR3. With regard to dermal fibroblasts and SSc, the potential TLR3 ligands are unknown. While viral triggers can be considered, there are no consistent associations of SSc with specific viral infections. It is intriguing to hypothesize that complexes of self-RNA andantimicrobial peptides, which have been reported to stimulate TLR7 and TLR8 [23], could also activate TLR3, but this is speculative. One additional hypothesis is that the ECM itself may serve as a TLR3 ligand. Indeed, in addition to PAMPs, TLRs can be activated by damage-associated molecular patterns (DAMPs). DAMPs are proinflammatory molecules generated upon tissue injury that include those released from necrotic cells as well as from the ECM. Tenascin-C has recently been reported to activate TLR4 during the development of inflammatory arthritis [24]. In the current study, the expression of TLR3 in human skin was demonstrated on dermal fibroblasts within dense connective tissue of the dermis. It is intriguing to hypothesize that the ECM may contain TLR3 ligands that could activate the dermal fibroblasts, even in the absence of a viral trigger.

The function of TLRs is best characterized in the innate immune system, where TLRs signal the presence of an infection and direct the adaptive immune response against microbial antigens [16]. The role of TLR signaling in fibroblasts is not as clearly understood. TLR stimulation of different fibroblast populations has been demonstrated to increase the production of chemokines and cytokines by fibroblasts, which subsequently can increase the inflammatory infiltration of the tissue. In this study, IFNα2 upregulated TLR3 and TLR3-induced IL-6 production. The increase in IL-6 could contribute to dermal fibrosis through increased fibroblast survival and proliferation, ECM deposition and myofibroblast differentiation [25-27]. In addition, IL-6 may act synergistically with TGF-β with regard to the development of tissue fibrosis [28]. Last, TLR3 activation may also directly regulate the behavior of fibroblasts. A recent report has demonstrated that TLR3 activation with poly(I:C) increased ECM and α-smooth muscle actin production, a marker of myofibroblast differentiation, by lung fibroblasts [29]. Together the effects of TLR3 directly on dermal fibroblast ability to differentiate into a myofibroblast and through the production of IL-6 may contribute to the development of dermal fibrosis.

Several independent studies have demonstrated that the type I IFN pathways are upregulated in patients with SSc compared with healthy controls [10-15]. However, the role of type I IFNs in the pathogenesis of SSc remains to be determined. Plasmacytoid dendritic cells (pDCs) are the primary source of type I IFNs in SLE [10,30]. It also has been suggested that pDCs are key producers of type I IFNs in SSc [31,32]. Type I IFNs subsequently regulate the behavior of key cells involved in the development of SSc, including dendritic cells, T-cells and dermal fibroblasts. This regulation of dermal fibroblasts could potentially be a pathologic or a protective response. In contrast to Th2 cytokines IL-4 and IL-13, which are profibrotic, type II IFNs such as IFN-γ decrease collagen production by dermal fibroblasts [33-37]. Type I IFNs have also been reported to decrease collagen production by dermal fibroblasts *in vitro *[35,36]. Consistent with the *in vitro *effects of IFNα2 on collagen production, administration of IFN-γ to mice decreased dermal fibrosis and collagen deposition in the bleomycin-induced skin fibrosis model [38]. However, clinical trials of recombinant IFN-γ or IFN-α in patients with SSc failed to show substantial clinical benefit [39-41]. The lack of effect of IFNs in SSc may be due to the timing of administration, the particular preparations of IFNs, pharmacokinetics or other clinical reasons. Alternatively, type I IFNs may have additional effects on the behavior of dermal fibroblasts that are independent of their antifibrotic properties.

The data presented herein suggest that type I IFNs may increase the inflammatory potential of the dermal fibroblast in part through the upregulation of TLR3 expression. Furthermore, IFNα2 increases the inflammatory potential more in SSc fibroblasts than in normal fibroblasts. We observed these effects at concentrations as low as 1 ng/mL IFNα2. The levels of IFNα2 within the microenvironment of the skin are not known. Therefore, it remains possible that the levels of IFNα2 used in the current study are higher than those found *in vivo*. At concentrations capable of inducing TLR3 expression, IFNα2 only marginally blunted TGF-β-induced collagen production, which itself was still significantly elevated relative to unstimulated dermal fibroblasts. Interestingly, it has recently been reported that TLR3 stimulation of dermal fibroblasts increased the expression of IFNα2- and TGF-β-responsive genes and that mice treated with subcutaneous TLR3 agonists developed dermal inflammation followed by fibrosis [42]. Together these observations suggest that IFNs may contribute to the development of SSc in a stepwise model wherein the pDCs produce type I IFNs, which regulate not only inflammatory cells but also dermal fibroblasts. Type I IFNs might then increase the expression of a number of molecules on the dermal fibroblast, including TLR3. TLR3 activation, either through viruses or through DAMPs, could increase the inflammatory potential of the dermal fibroblast, including increased IL-6 production, and could further increase IFN- and TGF-β-responsive gene expression. Together it is possible that the net balance would ultimately lead to the development of dermal inflammation and fibrosis. *In vivo *mouse studies will be helpful in determining the overall balance between the antifibrotic and proinflammatory properties of IFNs.

## Conclusions

In summary, our observations suggest that type I IFNs can increase the inflammatory potential of the dermal fibroblast through upregulation of TLR3 and its downstream responses. These studies add to our understanding of how type I IFNs, which are increased in SSc, may contribute to the pathogenesis of SSc. Additional studies are needed to further clarify how type I IFNs may contribute to SSc pathogenesis and to help determine whether type I IFNs can be a rational therapeutic target in SSc.

## Abbreviations

DAMPs: damage-associated molecular patterns; ECM: extracellular matrix; IFN: interferon; SLE: systemic lupus erythematosus; SSc: systemic sclerosis; TLR: Toll-like receptor.

## Competing interests

The authors declare that they have no competing interests.

## Authors' contributions

SKA, MW and FKT contributed to the study design, data acquisition, data analysis and interpretation, and manuscript preparation. CKL, DHP, MDM and FCA contributed to data acquisition and manuscript preparation.
